# Evaluation of the Value of Intraoperative Peri-Pancreatic Fluid Amylase Concentration in Predicting a Postoperative Pancreatic Fistula After Pancreaticoduodenectomy

**DOI:** 10.7759/cureus.44475

**Published:** 2023-08-31

**Authors:** Sravanti Balaga, Venkatarami Reddy Vutukuru, Sivaramakrishna Gavini, Chandramaliteeswaran Chandrakasan, Brahmeswara Rao Musunuru

**Affiliations:** 1 Department of Surgical Gastroenterology, Sri Venkateswara Institute of Medical Sciences, Tirupati, IND

**Keywords:** intraoperative amylase concentration, popf, ioac, amylase, pancreaticoduodenectomy, predictor of pancreatic fistula, pancreatic fistula

## Abstract

Background: Postoperative pancreatic fistula (POPF) is a common complication after pancreaticoduodenectomy (PD) and is a cause of significant morbidity and mortality. This study aimed to assess the predictive value of the amylase concentration of fluid accumulating in the peri-pancreatic region intraoperatively or intraoperative amylase concentration (IOAC) for the development of a clinically relevant POPF after PD.

Methods: All consecutive patients who underwent PD between April 2018 and May 2021 were prospectively included in the study. IOAC and postoperative day-three drain fluid amylase values were measured, and the incidence of clinically relevant POPF (CR-POPF) was noted. Receiver operating characteristic analysis was used to evaluate the predictive capacity of the IOAC for a CR-POPF.

Results: The study included 64 patients. A clinically relevant POPF was seen in 12 (18.8%) patients. On ROC analysis, the area under the curve (AUC) was 0.912 (with 95% CI of 0.822-1.001, p<0.001), which is highly significant. A cut-off IOAC value of 236 IU/L was derived, and an IOAC above this value was shown to predict the development of a CR-POPF in the postoperative period with a sensitivity of 91.7%. The highest positive predictive value (87.5%) was obtained with a cut-off of 772 IU/L.

Conclusion: An IOAC is an early, simple, and sensitive predictor for the development of a clinically relevant POPF after PD and can potentially aid in managing the resulting morbidity with intraoperative and postoperative measures.

## Introduction

Postoperative pancreatic fistula (POPF) is a severe and potentially life-threatening complication of pancreaticoduodenectomy (PD) [1,2]. It would, hence, be advantageous to predict the occurrence of a fistula as early as possible so that these patients may be monitored with greater vigilance, and the necessary interventions to lessen the morbidity of a fistula can be initiated sooner. 

Though a pancreatic fistula may take more than a week to manifest clinically, recent studies have shown that drain fluid amylase concentration on the first postoperative day itself can predict a fistula [3,4]. This suggests that a fistula occurs much earlier than previously believed. Considering that the leakage of pancreatic enzymes from the anastomotic site starts immediately after the anastomosis is done and even before epithelialization has occurred, thereby probably hampering the process of epithelialization, a more recently proposed theory suggests that amylase concentration in the fluid that collects around the pancreatico-enteric anastomosis during surgery; that is, intra-operative amylase concentration (IOAC) can be used to predict the occurrence of a pancreatic fistula [5]. We have undertaken this study to test this hypothesis in our practice with the belief that we may be able to alleviate the morbidity of a fistula by taking early measures if we are already expecting it. Our primary aim was to assess the predictive value of the IOAC after PD for the development of a clinically relevant POPF (CR-POPF) [6]. Our secondary aim was to determine the threshold value of the IOAC for predicting a CR-POPF.

This article was presented as an e-poster at the International Hepatopancreatobiliary Association (IHPBA)'s World Congress 2022 held in New York from March 30-April 2, 2022, and, thereafter, published in the compilation of abstracts presented at the conference in HPB journal (HPB. Oct 2022. 24:S380, DOI: 10.1016/j.hpb.2022.05.804).

## Materials and methods

A prospective observational study was conducted in our department at a tertiary care hospital in India between April 2018 and May 2021. All consecutive patients who underwent PD for any indication during this period were prospectively included in the study. There were no exclusion criteria. Approval was obtained from the Institutional Research and Ethics Committee.

Surgical technique and collection of peri-pancreatic fluid

PD with antral resection was done in all cases. The duct-to-mucosa technique was used for pancreaticojejunostomy, and it was done in two layers - mucosa to mucosa followed by pancreatic parenchyma to jejunal seromuscular layer. An internal stent was placed when the pancreatic duct diameter was less than 3 mm. Hepaticojejunostomy was done in a single layer of interrupted end-to-side sutures. Gastrojejunostomy was performed in a hand-sewn, side-to-side, antecolic isoperistaltic fashion in two continuous layers.

After the resection of the specimen and the first two anastomoses, i.e., pancreaticojejunostomy and hepaticojejunostomy, were completed, and the peri-pancreatic space was irrigated with 200 mL of normal saline. This irrigation fluid was suctioned and disposed of, and the area was mopped. Then, gastrojejunostomy was performed, taking care not to let enteral contents contaminate the field, and during the time that was taken to perform it (typically 20-25 minutes), out of whatever fluid had collected newly around the pancreatico-jejunal anastomosis, 3 mL of fluid was aspirated and sent to the lab to analyze its amylase concentration. This value was recorded as the IOAC [5]. Feeding jejunostomy was done in all cases to supplement enteral feeding till patients could consume adequate oral feeds and as a safety measure in case of occurrence of postoperative complications that could preclude oral intake. Two open abdominal drains were placed, one near the pancreaticojejunostomy and the other near the hepaticojejunostomy, which were used for assessing drain fluid amylase concentrations in the postoperative period.

Data collection

Along with patient demographics and lab investigations, intra-operative parameters comprising consistency of the pancreas, size of the pancreatic duct, and any postoperative complications, including 30-day mortality, were noted. Postoperatively, all patients with a normal clinical course were started on jejunostomy feeds from POD-2, RT was removed on POD-2 or POD-3, oral liquids were given from POD-4 or POD-5, and diet after two days, and patients were mostly discharged by POD-8 unless they required longer stay due to complications.

Bilateral drains’ fluid amylase levels and serum amylase levels were measured on postoperative days three and five. Drains were removed if the amylase level was less than 100 IU/L and daily drain fluid effluent was less than 100 mL. The postoperative day on which each drain was removed was noted. Other data pertaining to the management of a POPF were recorded as necessary. A POPF was defined and graded according to the revised International Study Group of Pancreatic Surgery (ISGPS) definition (2016) [6], considering Grade B and C fistulae as clinically relevant (CR-POPF).

Statistical analysis

The collected data were represented using numbers and percentages. For continuous variables, data were presented as mean +/- SD or median and range as appropriate. Comparison of parameters across groups was made by a t-test or ANOVA; non-parametric methods were used where required. The ability of the IOAC to predict the occurrence of a CR-POPF was assessed with the help of receiver operating characteristic (ROC) analysis as represented by the area under the curve (AUC). An AUC of more than 0.5 is considered significant, and the closer it is to 1, the better the correlation between the parameters. The optimal cut-off point for predictive IOAC was determined using sensitivity and specificity predictions at each cut-off value and finding the value with optimal sensitivity and specificity that can provide practical utility. Statistical software Statistical Product and Service Solutions (SPSS) version 16.0 (SPSS Statistics for Windows, Armonk, NY) and the MedCalc online software tool were used for statistical analysis.

## Results

A total of 64 PDs performed during the study period were included. The mean age of the patients was 54.98 years (±12.64 SD). There was a male predominance (64.1%) in the study population. Twenty-four (37.5%) patients had comorbid conditions, the most common being diabetes mellitus (seen in 20 patients). Eight of those 24 patients had multiple comorbid conditions. Most patients in our study were in the normal range of body mass index (BMI), with a mean of 21.41 kg/m^2^.

Incidence and management of a POPF

A clinically relevant pancreatic fistula (CR-POPF) was observed in 12 patients (18.8% incidence), out of which Grade B fistula (i.e., requiring minor interventions) was seen in 11 patients (17.2% of total) and Grade C fistula (i.e., requiring major interventions including surgery) was seen in one patient (1.6% of the total). An additional 19 patients (29.7% of the total) had only a biochemical leak (formerly called Grade A fistula; i.e., raised POD-3 drain fluid amylase values with normal clinical courses). All these patients with biochemical leaks had normal drain fluid amylase concentrations on POD-5, with low daily drain output (less than 20 mL); hence, their drains were removed during the hospital stay, and all these patients were discharged uneventfully.

Among the patients with Grade B fistula, one patient underwent image-guided percutaneous drain insertion for an intra-abdominal fluid collection; the remaining 10 patients had delayed removal of the abdominal drains (i.e., beyond three weeks). Out of these 10 patients, two patients also had surgical site infections, which were managed conservatively, and a third patient had Grade B delayed gastric emptying, in addition to the pancreatic fistula. The patient with the Grade C fistula had bleeding through the left drain and the surgical wound from POD-5, which could not be managed medically. He underwent reoperation on POD-7. While no active bleeding site could be identified, abdominal lavage was done, and drains were replaced. The patient had no further complications and was discharged 10 days after reoperation.

Patient demographics and operative and postoperative parameters

We have divided the study population into two sub-groups for comparison, i.e., the "fistula group" (n=12) and the "no fistula group" (n=52). Various characteristics, including predictive factors for a POPF in patients of the two groups, have been compiled in Table 1. Demographics and lab investigations were comparable between both groups. Mean BMI was lower in the "no fistula" group, but both groups were in the normal range. Among intraoperative predictive factors, the number of patients with pancreatic duct diameter less than 3 mm was significantly higher in the "fistula" group (p-value of 0.014). The postoperative histopathology in both groups had a similar distribution (p-value of 0.243), the most common being ampullary adenocarcinoma in both the "fistula" (41.7%) and "no fistula" (53.8%) groups. Pancreatic adenocarcinoma accounted for 25% and 17.3% of patients, respectively. Non-malignant pathologies were seen in 8.3% of the "fistula" group (one patient each with solid pseudopapillary neoplasm and low-grade duodenal GIST) and 9.5% in the "no fistula" group (solid pseudopapillary neoplasm in two patients, chronic pancreatitis in two patients and tuberculosis of pancreas in one patient), respectively.

**Table 1:** Analysis of patient characteristics and postoperative pancreatic fistula predictive factors

	Total (n=64)	Fistula group (n=12)	No fistula group (n=52)	p-value
Age in years (Mean ± SD)	54.98 ± 12.64	51.17 ± 11.33	55.87 ± 12.85	0.249
Sex (M: F)	41: 23	6:3	32: 20	0.381
BMI (Mean±SD) (kg/m^2^)	21.41 ± 3.92	23.36 ± 3.81	20.95 ± 3.84	0.55
Comorbid conditions	37.5%	58.3%	32.7%	0.098
Hemoglobin (Mean±SD) (gm/dl)	10.78 ± 2.06	10.84 ± 3.07	10.77 ± 1.80	0.916
Serum albumin (Mean±SD) (gm/dl)	3.36 ± 0.46	3.37 ± 0.51	3.36 ± 0.45	0.973
Soft pancreas	15 (23.4%)	5 (41.7%)	10 (19.2%)	0.202
Duct diameter <3 mm	23 (35.9%)	8 (66.6%)	14 (28.6%)	0.014
Median IOAC (IU/L)	163	873.5	105	<0.001
IOAC >236 IU/L	27 (42.2%)	11 (91.66%)	16 (30.77%)	<0.001
Hospital stay (Mean±SD) (Days)	12.16 ± 5.35	13.67 ± 3.968	12.27 ± 5.754	0.488
30-day mortality	0	0	1	0

Even though the incidence of all postoperative complications combined was higher in the "fistula" group, it was not statistically significant (Table 2). The overall incidence of delayed gastric emptying (DGE) was 15.62% (10 patients), and that of postpancreatectomy hemorrhage (PPH) was 6.25% (four patients). Among the "fistula" and "no fistula" groups, DGE was seen in 25% and 13.46% (p-value of 0.321), PPH in 8.33% and 5.77% (p-value of 0.741), and wound infection in 33.33% and 17.31% (p-value of 0.214) of patients, respectively.

**Table 2:** Postoperative complications

Incidence	Total (n=64)		Fistula group (n=12)		No fistula group (n=52)		p-value
DGE	10	15.62%	3	25%	7	13.46%	0.321
PPH	4	6.25%	1	8.33%	3	5.77%	0.741
Wound infection	13	20.31%	4	33.33%	9	17.31%	0.214

There was no significant difference in the mean hospital stay of patients between the groups (p=0.853). There was a single mortality in the "no fistula" group due to cardiac morbidity. There was no POPF-related or other 30-day mortality in the "fistula" group.

IOAC and pancreatic fistula

The IOAC values in the study population ranged from 10 IU/L to 1983 IU/L, with a median of 163 IU/L. Among the patients in the "fistula" group, IOAC values ranged between 129 IU/L and 1983 IU/L, with a median of 873.5 IU/L. In the "no fistula" group, the IOAC ranged between 10 IU/L and 782 IU/L, with a median of 105 IU/L. This difference was highly significant, with a p-value of <0.001.

ROC Curve Analysis of the IOAC

**Figure 1:**
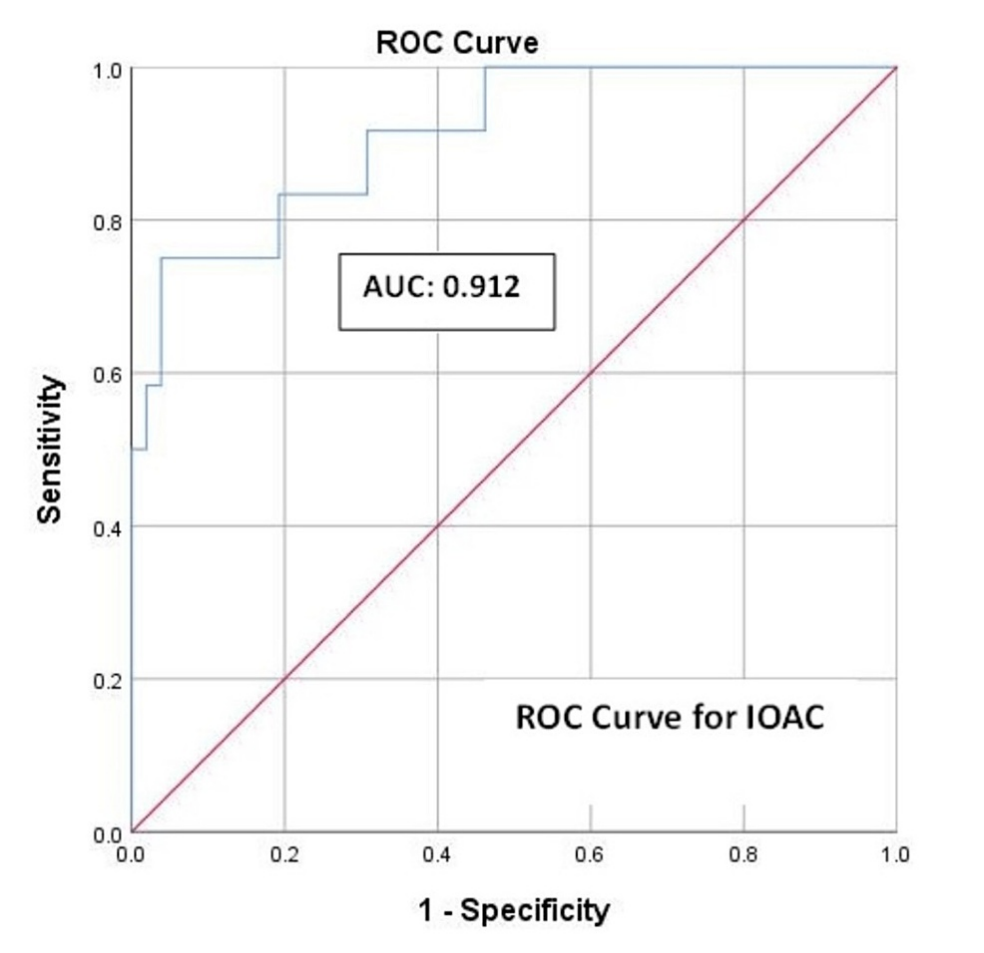
ROC curve for the prediction of a CR-POPF using the IOAC

The ROC curve for analyzing the predictive capacity of the IOAC for a POPF in our study is depicted in Figure 1. The AUC was 0.912 with a 95%CI of 0.822-1.000 (standard error: 0.046) and a p-value of <0.001. An AUC of >0.75 is considered accurate for clinical prediction; hence, our result was highly significant. As per this analysis, the optimal cutoff or threshold value of the IOAC that can predict a fistula was selected to obtain the highest sensitivity with acceptable specificity (with the lowest false-negative rate of 8.33%), i.e., IOAC of 236 IU/L (Youden index of 0.60, sensitivity of 91.7%, specificity of 69.2%, negative predictive value (NPV) of 97.3%, and positive predictive value (PPV) of 40.74%). However, the cutoff value with the highest Youden index (0.70) was 648.50 IU/L, which had a sensitivity of 75%, specificity of 96.15%, PPV of 81.8%, and NPV of 94.3%. The highest positive predictive value was obtained with an IOAC cutoff of 772 IU/L (with 87.5% of PPV, 91.07% of NPV, sensitivity of 58.33%, and specificity of 98.08%).

IOAC vs. POD-3 Drain Fluid Amylase Values

Overall, 48.4% of patients (i.e., 31 out of 64 patients) had elevated amylase concentrations in their drain fluid on POD-3 (i.e., more than three times the upper limit of the institutional normal serum amylase value). Among these patients, the majority (i.e., 19; 61.29% of patients) had a normal clinical course, hence considered to have a biochemical leak. The remaining 12 patients (38.71%) had a CR-POPF (including the one patient with an IOAC lower than 236 IU/L). None of the patients with a normal day three drain amylase value had a CR-POPF (i.e., no latent fistulae). Thus, in our study, the POD-3 drain amylase value had a sensitivity of 100% and a specificity of 63.46%, a positive predictive value of 38.71%, and a negative predictive value of 100%, to predict a CR-POPF.

## Discussion

A POPF is a dreaded cause of morbidity and mortality after PD. The ISGPS defines a clinically relevant POPF as any measurable volume of output in the drain on or after postoperative day three with an amylase level more than three times the upper limit of the normal institutional serum amylase value [6]. 

A POPF itself may lead to highly morbid sequelae such as intra-abdominal hemorrhage or collections with sepsis. It may also increase the possibility of other complications such as delayed gastric emptying, bile leak, prolonged hospital stay, and mortality [7]. Owing to the severity of complications that a pancreatic fistula can lead to, it would be beneficial for the surgeon to identify patients at risk of developing a fistula or even to detect a fistula early. 

A variety of strategies have been studied to prevent or reduce the morbidity of a fistula, such as the use of octreotide [8], pasireotide [9], delayed enteral feeds [7], restriction of the intravenous fluid regimen, increased use of hypertonic intravenous fluids [10], delayed removal of abdominal drains [11], and recently administrating trypsin inhibitors such as ulinastatin [12], etc.

Thus, identifying high-risk patients early in the postoperative period would help us manage them suitably, with intensive care and increased vigilance, thereby catching leaks early and mitigating their morbidity and possible mortality, reducing hospital stay, and, as a result, improving the quality of life of patients. A lower threshold for repeat imaging and intervention or re-exploration may benefit patients with a high risk for a POPF.

The current definition of a POPF considers a high drain fluid amylase concentration on postoperative day three [6]. However, some patients who have normal drain fluid amylase values (and may hence even undergo removal of drains) have been known to develop a fistula afterward (a latent fistula) [13]. Additionally, a majority of patients with high drain fluid amylase values on day three do not develop a clinically relevant fistula [14,15] in their postoperative course; that is, they only have a biochemical leak and are clinically stable. Hence, the relevance of the day-three drain amylase value in defining or predicting a fistula is in itself questionable.

de Reuver et al. proposed in their study that the integrity of the pancreatico-enteric anastomosis is determined at the time of suturing itself, and any leak from the anastomosis should be amenable to detection immediately after forging the anastomosis [5]. Wada et al. showed that using a surgical microscope during the creation of the anastomosis was associated with a lesser chance of a pancreatic leak [16]. This finding also strengthens the argument that the quality of the anastomosis is reflective of the risk of occurrence of a fistula. Two groups have tested this theory for predicting a POPF after pancreaticoduodenectomy [5] and after distal pancreatectomy [17], respectively, which showed a significant association of the IOAC with postoperative outcomes, namely, pancreatic fistula, delayed gastric emptying, post-pancreatectomy hemorrhage, and Grade IV & V complications as per the Clavien-Dindo classification. Another study by Wang et al. for distal pancreatectomy also demonstrated the reliability of the IOAC for predicting a POPF [18].

In the present era, where the prudence of abdominal drain placement is being questioned [11,19,20], and early drain removal is advocated even when drains are placed [11,21-23], intra-operative fluid amylase measurement may emerge as a better alternative to drain fluid amylase assessment. Theoretically, numerous possible benefits can be derived by using the IOAC, namely, the prospect of revision/scaffolding of the pancreatico-jejunal anastomosis during the primary procedure itself (if fluid amylase values can be obtained from the lab before abdominal closure, i.e., within one hour of sampling), avoidance of abdominal drains outright in patients with low IOAC, and postoperative patient stratification (i.e., assigning enhanced recovery protocols to patients with a low IOAC, while patients with a high IOAC may undergo stringent monitoring with a lower threshold for further investigation and intervention). Thus, we may be able to optimize patient care and reduce unnecessary costs during the perioperative period.

In our study, we have found that the IOAC predicts the occurrence of a clinically relevant POPF after pancreaticoduodenectomy (Grade B and C POPF as per the revised ISGPS classification) [6] with nearly 91.7% sensitivity when the cutoff is 236 IU/L, but at the cost of a low positive predictive value of 40.74%. When the cutoff is raised to 772 IU/L, the positive predictive value increases significantly to 87.50%. Hence, we propose that the lower cutoff value of 236 IU/L may be used to select patients who need greater perioperative vigilance and implementation of postoperative measures, including estimation of drain fluid amylase values on days one, three, and five, administration of octreotide; delaying oral feeds; etc. An IOAC above the higher cutoff value of 772 IU/L may prompt the surgeon to assess if intra-operative interventions, such as revision or scaffolding of anastomosis, would be beneficial in preventing a fistula. Patients with an IOAC lower than 236 IU/L may be allotted enhanced recovery protocols, including complete avoidance of drains or early drain removal.

The limitations of our study are as follows: the small sample size and, thus, the inability to perform a sub-group analysis with stratification of risk of each grade of fistula (B, C) separately; a possibility of error in collecting the sample, such as dilution or contamination; time taken to create the gastrojejunostomy may affect the amount of pancreatic fluid leaking from the anastomosis and act as a confounding factor, and, hence, violation of a predetermined time limit may need to be considered as an exclusion criterion; the cutoff IOAC of 236 IU/L to predict a CR-POPF has high sensitivity at the cost of a low positive predictive value; and the IOAC in our study had a false-negative rate of 8.33%, so some patients who actually develop a fistula later may not receive stricter peri-operative surveillance as they have a low IOAC. However, the last limitation is also encountered when POD-3 drain fluid amylase is used to predict a fistula [13].

## Conclusions

We conclude that the IOAC is a sensitive intra-operative predictor for the development of clinically relevant pancreatic fistula after PD. We suggest that it has potential utility in reducing the incidence or severity of pancreatic fistulae by enabling the use of intraoperative and postoperative interventions and, hence, is a useful addition to the armamentarium of pancreatic surgeons. We hope our study serves as an impetus for further research on this inexpensive and simple predictor. Large-volume studies are required to validate the findings of our study, to deduce an optimal cutoff value of the IOAC that can predict a fistula, and to evaluate the utility of interventions in preventing pancreatic fistula in patients with a high IOAC.
